# Foreign Body with Gas Gangrene in an Elderly Patient with Diabetes

**DOI:** 10.4172/2155-6156.1000310

**Published:** 2013-11-27

**Authors:** Sherley Abraham, Erica Hall, Sherita Hill Golden, Rita Rastogi Kalyani

**Affiliations:** Division of Endocrinology, Diabetes & Metabolism, Johns Hopkins University School of Medicine, USA

**Keywords:** Diabetes, Gangrene, Infection, Foreign body, Neuropathy, Elderly

## Case Report

A 76-year-old African-American man who lived alone, with an 11-year history of poorly controlled diabetes and symptomatic peripheral neuropathy, presented to the Emergency Department with right foot pain and swelling. His initial evaluation included a lower extremity Doppler that ruled out a deep venous thrombosis and the patient was discharged. The patient returned to the Emergency Department after one week for worsening right foot pain and swelling. He was afebrile and hemodynamically stable. The physical exam revealed new unroofed blisters on the dorsal aspect of the right foot. Foot x-ray (Panel 1) showed a 3 cm linear metallic foreign body, which appeared to be a broken sewing needle in the soft tissues between distal first and second metatarsals. Computed Tomography (CT) scan (Panel 2) showed moderate air suggesting deep tissue infection, with multiple tiny gas bubbles within the proximal phalanx of the second digit, concerning for gas gangrene. He received broad-spectrum intravenous antibiotics and underwent a two-stage operation on his right leg. The first stage was a guillotine amputation. The patient remained afebrile and hemodynamically stable. Antibiotics were stopped since now, after removal of the gangrenous tissue, there was adequate source control of the infection. A few days later, he underwent a definitive, below-the-knee amputation.

The differential diagnoses for gas gangrene includes inflammation of any of the various tissue layers, from the skin to the deep tissues and bone such as cellulitis, deep venous thrombosis and thrombophlebitis, necrotizing fasciitis, myositis, rhabdomyolysis, or osteomyelitis [1,2]. In diabetic foot infections, imaging with plain films of the foot and ankle can be used to evaluate for foreign bodies, soft tissue gas, bony destruction or deformity [1]. CT is considered the imaging modality of choice when evaluating for gas or emphysematous infections. In cases of gangrene, CT can be used to confirm the diagnosis, determine the anatomic location and extent of the infection, and for follow-up after treatment [3]. There have also been cases of abdominal infections leading to gangrene in the limbs. In these cases, CT was especially useful in determining the source of infection [4,5]. In diabetic foot infections, if there is suspicion of bone infection, MRI is preferred to confirm the diagnosis of osteomyelitis [1]. Evidence of subcutaneous gas on imaging, as well as the presence of other physical findings including bullae, ecchymosis, and skin necrosis, suggest necrotizing soft tissue infection which requires surgical evaluation [1]. Without early imaging to elucidate the diagnosis, gas gangrene may rapidly progress systemically to sepsis, shock, or even death [2].

Gas gangrene is a potentially life threatening infection, and requires emergent surgical intervention for definitive treatment. Medical therapies, including broad-spectrum antibiotics, are important treatment modalities in addition to surgery, but the antibiotics may not penetrate adequately to the ischemic tissue [2]. The definitive treatment of gas gangrene of the foot is a two-stage surgery [6]. Guillotine amputation is a procedure in which all of the tissues from the skin to the bone are cut at the same level, without the creation of soft tissue flaps. These procedures are performed in cases of severe infection or necrosis. The guillotine amputation is used as a first stage to control the infection and bacteremia. The second stage is definitive surgery, with below the knee amputation, which is performed in a controlled wound environment. In the second stage, the higher level amputation and creation of soft tissue flaps will cover the open end of the stump [6].

Among U.S. adults with diabetes, the prevalence of any lower extremity disease (including peripheral neuropathy, peripheral arterial disease, foot ulcers, and lower extremity amputations) is twice as high as individuals without diabetes [7]. Non-traumatic lower extremity amputation disproportionately affects individuals with diabetes who are elderly (≥75 years old), African-Americans, and men [8]. Asymmetric foot swelling in a patient with diabetes and severe neuropathy should prompt further radiographic evaluation to potentially identify a foreign body and prevent serious infection, even in the absence of fever.

Our case highlights the importance of early imaging to diagnose foreign body with gas gangrene in a high-risk patient with diabetes and to prevent potentially fatal complications. In other reports of patients with diabetes and gas gangrene who were not considered surgical candidates due to multiple comorbidities, antibiotic treatment alone was inadequate in halting the spread of infection, leading to bacteremia and death [9]. Ultimately, a comprehensive diagnostic and radiographic evaluation in a patient with diabetes and asymmetric foot swelling may prevent the loss of limb and functional independence, especially in those at high-risk for physical disability such as the elderly [10].

## Figures and Tables

**Panel 1:**
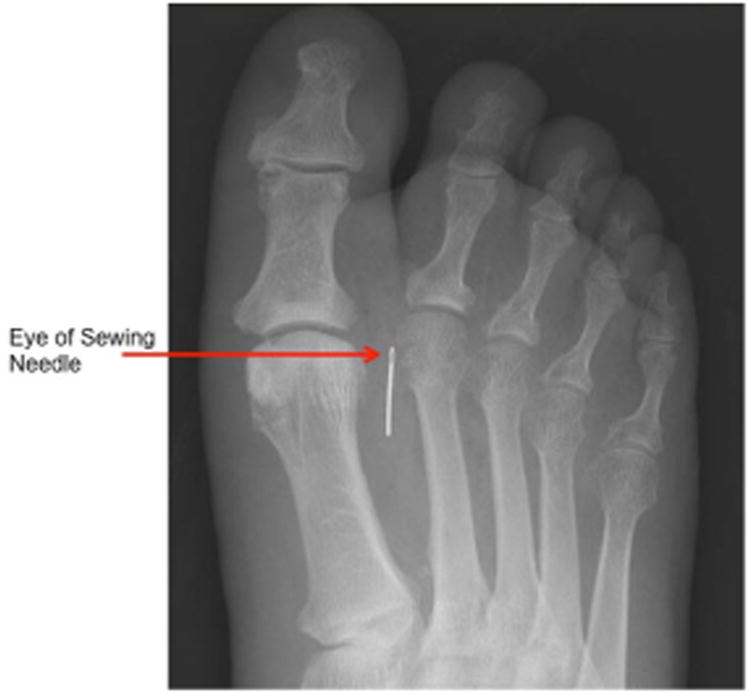
Foot X-ray shows a 3 cm linear metallic foreign body, a broken sewing needle in the soft tissues between distal first and second metatarsals.

**Panel 2:**
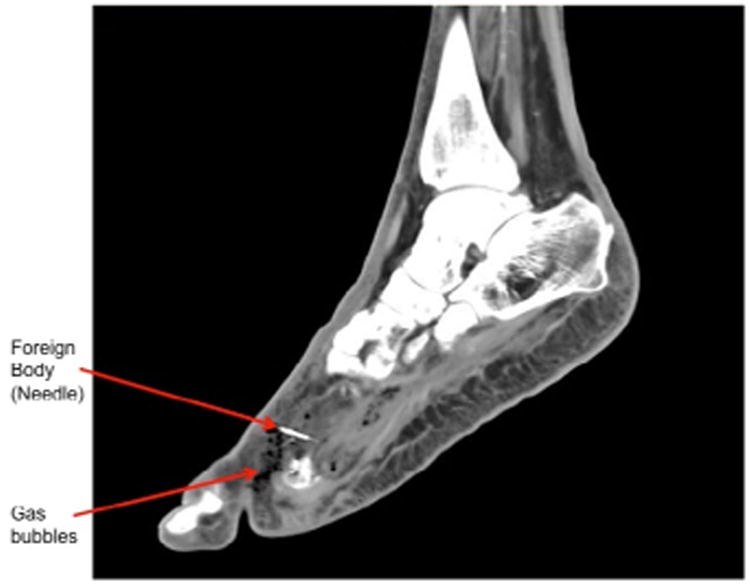
CT scan shows moderate air suggesting deep tissue infection with multiple tiny gas bubbles.

## Learning Points

- Individuals with diabetes who are elderly (≥75 years old), African-Americans, and male, are at a disproportionately high risk for non-traumatic lower extremity amputation.
- The incidence of peripheral neuropathy and peripheral arterial disease are two-fold higher in persons with diabetes. This increases the risk of foot ulcers, which if left untreated can lead to lower extremity amputation.
- In diabetic foot infections, appropriate imaging to identify the presence of foreign bodies, soft tissue gas, bony destruction or deformity, deep soft tissue infection or osteomyelitis is critical in the initial diagnostic evaluation.
- Gas gangrene requires early identification and emergent surgical intervention to prevent the rapid progression to septic shock.

## References

[1] Wukich DK, Armstrong DG, Attinger CE, Boulton AJ, Burns PR (2013). Inpatient management of diabetic foot disorders: a clinical guide. Diabetes Care.

[2] Shukla A, Rosen CL, Wong JK (2011). Emergent Treatment of Gas Gangrene. Medscape.

[3] Grayson DE, Abbott RM, Levy AD, Sherman PM (2002). Emphysematous infections of the abdomen and pelvis: a pictorial review. Radiographics.

[4] Wright WF (2012). Clostridium septicum myonecrosis presenting as an acute painful foot. Am J Emerg Med.

[5] Balicco B, Manzoni D, Ancora C (2009). A case of fatal emphysematous pyelonephritis presenting as lower limb gaseous gangrene. Minerva Anestesiol.

[6] Panchbhavi VK, Schraga ED (2013). Guillotine Ankle Amputation. Medscape.

[7] Gregg EW, Sorlie P, Paulose-Ram R, Gu Q, Eberhardt MS (2004). Prevalence of lower-extremity disease in the US adult population >=40 years of age with and without diabetes: 1999-2000 national health and nutrition examination survey. Diabetes Care.

[8] Li Y, Burrows NR, Gregg EW, Albright A, Geiss LS (2012). Declining rates of hospitalization for nontraumatic lower-extremity amputation in the diabetic population aged 40 years or older: U.S., 1988-2008. Diabetes Care.

[9] Ghosh S, Bal AM, Malik I, Collier A (2009). Fatal Morganella morganii bacteraemia in a diabetic patient with gas gangrene. J Med Microbiol.

[10] Kalyani RR, Saudek CD, Brancati FL, Selvin E (2010). Association of diabetes, comorbidities, and A1C with functional disability in older adults: results from the National Health and Nutrition Examination Survey (NHANES), 1999-2006. Diabetes Care.

